# Chronic Bickerstaff’s encephalitis with cognitive impairment, a reality?

**DOI:** 10.1186/1471-2377-14-99

**Published:** 2014-05-06

**Authors:** Mathilde Renaud, Jérôme Aupy, Guillaume Camuset, Nicolas Collongues, Jean-Baptiste Chanson, Jérôme de Seze, Frédéric Blanc

**Affiliations:** 1 University Hospital of Strasbourg, Neuropsychology Unit, Neurology Service and CMRR (Centre Mémoire de Ressources et de Recherche), 1 avenue Molière, 67098 Strasbourg Cedex, France; 2 University of Strasbourg and CNRS, ICube laboratory UMR 7357 and FMTS (Federation de Médecine Translationnelle de Strasbourg), team IMIS/Neurocrypto, Strasbourg, France; 3 University Hospital of Strasbourg, CMRR (Memory Resources and Research Centre), Strasbourg, France; 4 University of Strasbourg, INSERM U119 laboratory, Strasbourg, France; 5 University Hospital of la Réunion, Infectious Diseases Unit, Saint Pierre, La Réunion, France

**Keywords:** Bickerstaff’s encephalitis, Anti-ganglioside antibodies, Chronic encephalitis, *Campylobacter jejuni*, Molecular mimicry, Cognitive dysfunction, Dementia, Mild cognitive impairment

## Abstract

**Background:**

Bickerstaff’s encephalitis (BE) is an acute post-infectious demyelinating disease with albuminocytological dissociation. A chronic form has rarely been described previously.

**Case presentation:**

A 44-year-old man was hospitalized for drowsiness, cognitive complaint limb weakness, ataxia and sensory disturbance after diarrhea. Neuropsychological evaluation showed slowing, memory and executive function impairment, while analysis of the CSF showed albuminocytological dissociation. Immunologic tests showed positive anti-ganglioside antibodies (anti-GM1 IgM, anti-GD1a IgG and anti-GD1b IgM). Brain MRI was normal but SPECT showed bilateral temporal and frontal hypoperfusion. Outcome under immunoglobulin treatment (IVIG) was favorable with an initial improvement but was marked by worsening after a few weeks. Consequently, the patient was treated with IVIG every 2 months due to the recurrence of symptoms after 6 weeks.

**Conclusion:**

This case raises the question of the existence of a chronic form of BE with cognitive impairment, in the same way as chronic inflammatory demyelinating polyneuropathy is considered to be a chronic form of Guillain–Barré syndrome.

## Background

In 1951, Bickerstaff and Cloake described an encephalitis consisting in an association of ophthalmoplegia, ataxia and consciousness disturbance following an acute infection [1-4]. This entity known Bickerstaff’s encephalitis (BE) is close to Guillain–Barré syndrome (GBS) and Miller Fischer syndrome, with the presence of an albumino-cytological dissociation in the cerebrospinal fluid [2]. The etiology of BE is speculated to be similar to that of GBS because of the existence of a prodromal infection. Several studies have shown that anti-ganglioside antibodies can be positive in BE [4-6]. The course of BE is usually described as monophasic, with only one acute episode. The prognosis depends on the severity of consciousness disturbance, which can range from drowsiness to coma. Treatment consists in intravenous corticosteroids, intravenous immunoglobulin (IVIg) or plasmapheresis [5-7].

We report a case of a patient in whom the course was chronic with cognitive impairment, suggesting the existence of a chronic form of BE.

## Case presentation

A 44-year-old man, in whom the only antecedent was lumbar disc herniation, had experienced various symptoms while on a visit to Senegal. The symptoms that appeared one week after his arrival- included high fever, chills, drowsiness and not-bloody diarrhea. Two weeks after his return in France, he experienced additional symptoms: intense drowsiness with cognitive complaint, leg weakness, asthenia, joint pain (elbows, wrists, knees) and diffuse myalgia. Severe psychomotor slowing and impaired concentration led him to consult a physician. The patient was referred to a local hospital but no diagnosis was made at that time. After one year without a diagnosis and progressive worsening, he was examined at our tertiary center. He complained of drowsiness, cognitive difficulties, but also mild diarrhea. Clinical examination revealed a deficit in all four extremities, deep sensory impairment in the lower limbs, proprioceptive ataxia, distal pain, diminished cognitive speed and attention deficit. He presented also with a vitiligo. The rest of the physical examination was unremarkable: in particular, tendon reflexes were present and symmetrical and there were no abnormal eye movements. Neuropsychological evaluation found a diminished speed of information processing, short-term and working memory impairment and attention and executive functioning disorders: the patient had a “subcortical” cognitive impairment. Thus, we used the BCcogSEP battery to evaluate cognitive functions [8]. BCcogSEP is the French modified version of the Brief Repeatable Battery of Neuropsychological tests for Multiple Sclerosis (BRB-N) proposed by Rao and comprises the 5 modified BRB-N tests: a Selective Reminding Test (BCcog-SRT), a visuospatial memory test (10/36), the Paced Auditory Serial Addition Test (PASAT), a verbal fluency test, and the digit symbol substitution test of the WAIS-R (DSST) [9]. Three tasks were added in order to provide additional information about working memory and executive functions: the crossed tapping test, a “Go-No-Go” test and the WAIS-R digit span subtest. Among these 14 subtests, 11 were impaired, including the three subtests of SRT, the immediate recall of 10/36, the forward and backward digit span, the DSST, the PASAT, the crossed tapping test and the semantic and phonemic fluency tests.

Moreover, Free and Cued Selective Recall Reminding Test (FCSRT) [10] for verbal episodic memory showed a normal score of 16 out of 16 words for immediate recall and scores of 6-9-9/16 (<5th percentile) for free recall and 12-15-15/16 for total recall (i.e. within the normal range), showing an improvement after cueing. In the visual object recognition memory test (DMS 48) [11] the patient was deficient, with only 71% correct answers a few minutes after presentation of the pictures, and 69% correct answers one hour after. The Mini-Mental Status Examination (MMSE) score was normal: 28 (recall 1/3). The Rey-Osterrieth complex figure copy was normal [12].

**Figure 1:**
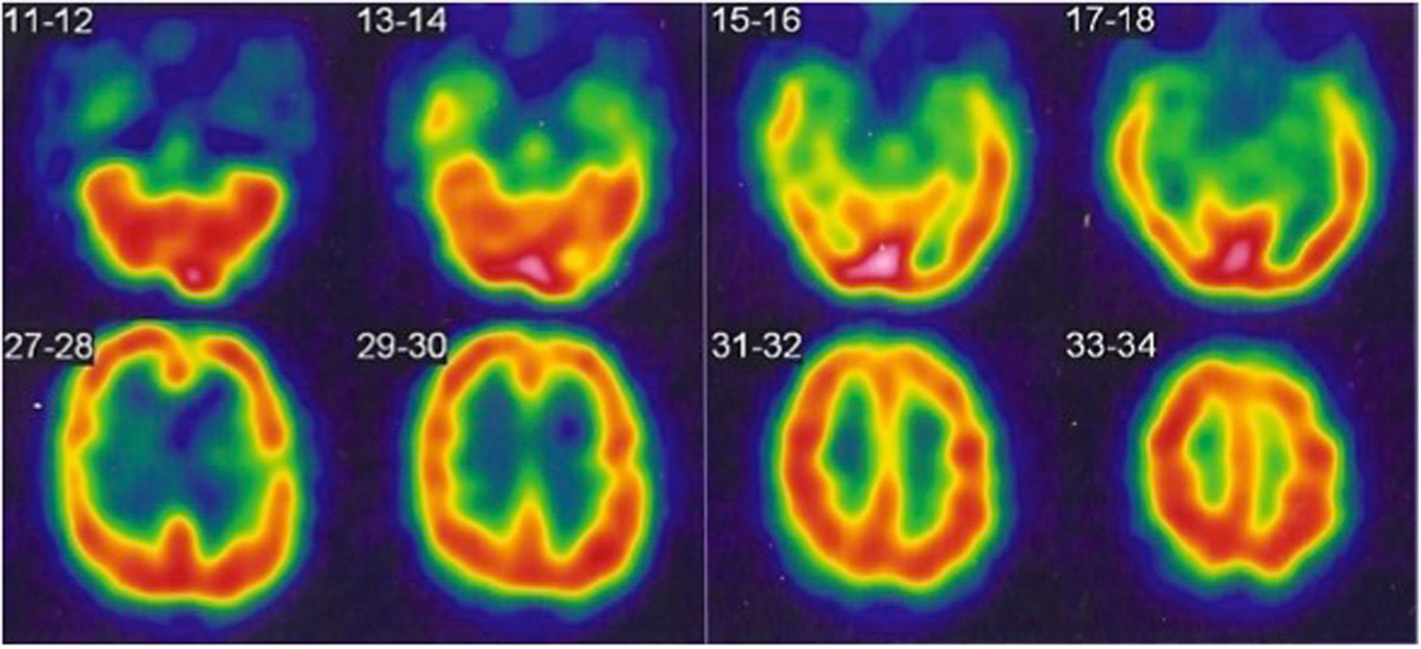
SPECT of the patient with chronic BE showing bilateral temporal and frontal hypoperfusion.

Brain MRI and electroencephalogram were normal whereas SPECT showed bilateral temporal and frontal hypoperfusion (Figure 1). Somatosensory evoked potentials (SEP) were abnormal in the left leg, with a discrete delayed cortical wave latency. The motor nerve conduction study revealed a right carpal tunnel syndrome but found no other abnormalities; in particular, there was no evidence to suggest demyelination or axonal degeneration.

The CSF study showed a high protein level (0.80 g/l) with a normal number of white and red cells, negative polymerase chain reaction results for herpes simplex virus, the absence of oligoclonal bands, and a negative Lyme serology. These findings indicated the presence of an albumino-cytological dissociation. Different infectious causes has been researched in the blood but were negative: HCV, HBV, HIV, anguillula, ascaris, trypanosoma, schistosoma, fasciola hepatica, malaria, tropheryma whipplei, blastocystis hominis. Stool parasitological examinations were negative. Bacteriological examination of stool samples (after the third taking) revealed the presence of *Campylobacter jejuni*. Anti-ganglioside antibodies were positive (immunodot; anti-GM1 IgM: 1/2000, anti-GD1a IgG: 1/2000, anti-GD1b IgM: 1/500, anti-GQ1b: negative).

BE was diagnosed and the patient was treated with IVIg for 5 days. This led to an improvement in symptoms (i.e. regression of limb weakness and pain, improvement in cognitive impairment, particularly in terms of language and memory).

Unfortunately, after 2 months, the patient relapsed with the same clinical presentation. This led us to consider his symptoms as chronic. Consequently, he received IVIG treatment every 2 months, with a systematic recurrence of symptoms after 6 weeks. An immunosuppressive drug, mycophenolate mofetil, was tested, but was poorly tolerated and the patient was switched to azathioprine 100/mg day that was also stopped because it did not permit to diminish the frequency of IVIG. For the past 4 years, the time between 2 courses of IVIG has been about 3 months. The patient is now stabilized with this treatment but it is impossible to stop IVIG permanently as a relapse occurs a few weeks after IVIG withdrawal. Nevertheless, the patient probably presented a chronic progressive form of BE because relapses are not totally stopped despite the treatment.

## Discussion

In our patient, the diagnosis of chronic BE was based on the following: sensory and motor disturbances in all four limbs, vigilance and cognitive disorders, CSF albuminocytological dissociation, positive anti-ganglioside antibodies, and presence of *C. jejuni* in stool samples. Chronic form of BE is suspected since the patient worsened before IVIG treatment and since every stop of the treatment pulls a worsening a few weeks later.

The classic presentation of BE, combining classic symptoms such as consciousness disturbance, ophthalmoplegia or ataxia, may be incomplete and/or associated with other neurological signs (Babinski’s sign, sensory disturbance, facial and limb weakness) [13].

The albuminocytological dissociation typically appears after the second week [14]. When anti-ganglioside antibodies are positive, they can be of various types: IgG antibody to GQ1b is the most frequent [6,13-16]. According to the study of Odaka et al. including 62 patients, [14], anti-GQ1b antibodies are positive in 66% of cases - and thus negative in 34% of cases-. It seems that antibodies to GD1a, GD1b and GM1 are more often positive when Bickerstaff’s encephalitis is associated with limb weakness as in our case [14]. Anti-gangliosides IgM antibodies (such as two antibodies for our patient) are usually associated with chronic forms of neuropathies [17,18].

*C. jejuni* is frequently involved in BE, as in our case where the primary symptom was diarrhea [13,14,16,19,20]. Interestingly, our patients has had a chronic diarrhea and developed a chronic form of BE. Recently, animal models of infection with campylobacter jejuni showed that long-term *C. jejuni* infection not only induced intestinal but also long term extra-intestinal immune responses in organs [21]. Indeed, mechanisms for induced autoimmunity by infection include the activation of autoreactive cells by an encounter with a pathogen epitope [22]. Molecular mimicry may trigger autoimmune tissue damage and induce acute inflammation, as in the case of GBS and Miller Fisher syndrome [23]. This acute form of inflammation could evolve into a chronic form [24].

The electroencephalogram is reported to be abnormal, showing slow activity in the theta or delta range in 57–70% of cases [13,14]. Electromyographic evidence of demyelination or axonal degeneration is found in half of all cases [13,14]. These two examinations were normal in our patient but they were performed more than a year after the onset of the disease.

Brain MRI is reported to be normal in 75% to 90% of patients with BE, as was the case in our patient [4,25-27]. Hyperintensities in the pons, thalamus, medulla oblongata or midbrain, corresponding to vasogenic edema, are sometimes observed. This vasogenic edema could be reversible and not visible in MRI.

Neuropsychological tests were not described in previous studies on BE. In our patient, the cognitive profile is fronto-temporal with slowing, which corresponds to the SPECT abnormalities. SPECT could be abnormal in BE while MRI is normal. Indeed, in a recent case report of BE with normal MRI, SPECT showed hypoperfusion of the whole cerebral hemispheres and basal ganglia with relative sparing of the thalami and of the brainstem [28]. SPECT might therefore be a diagnostic tool in BE despite a lack of specificity.

Immunotherapy (IVIg or plasmapheresis) is usually the standard treatment for BE [5,7,29]. In our patient, IVIg dependence suggests a progression to chronic autoimmune encephalitis with persistent inflammatory activity. BE is considered to be a monophasic disease. Only rarely is BE documented to relapse [30,31]. A patient, presented with recurrent drowsiness, unsteady gait, diplopia, has been described as recurrent Fisher-Bickerstaff syndrome by Dong et al. [30]. Sharma et al. described a second case with recurrent BE with overlapping features of Guillain-Barré and Miller-Fisher syndromes without anti-gangliosides [31]. Nonetheless, our case was different because he had rather a progressive chronic form without total relapses. Indeed, if we stop the IVIg, he deteriorates.

## Conclusions

**Figure 2:**
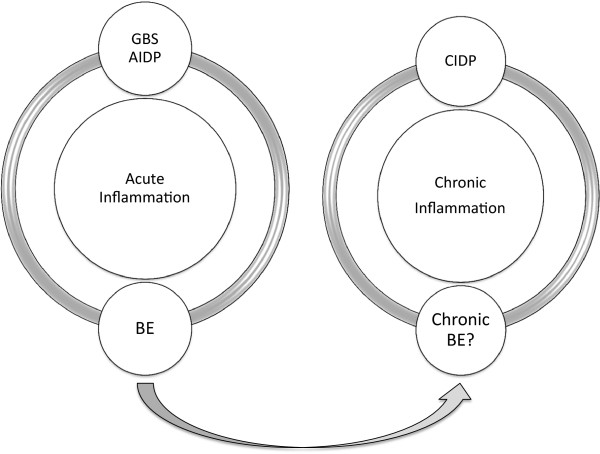
Plan of the supposed place of chronic BE within the acute and chronic inflammatory pathologies of the nervous system.

Our case suggests the existence of a possible chronic Bickerstaff’s encephalitis with anti-gangliosides, underpinned by mechanisms of molecular mimicry, secondary to infection, and then evolving on its own account like other neurological autoimmune diseases. Also, there is a parallelism and/or a continuum between some neurological inflammatory diseases: for example, between Guillain-Barré syndrome and chronic inflammatory demyelinating neuropathy (CIDP) (Figure 2). In conclusion, this case could correspond to a chronic form of BE.

## Consent

Written informed consent was obtained from the patient for publication of this case report and any accompanying images. A copy of the written consent is available for review by the Editor of this journal.

## Competing interests

The authors declare that they have no competing interests.

## Authors’ contributions

MR conceived of the case report. FB participated in its design and coordination and helped to draft the manuscript. JA, GC, NC, JBC, JDS read and approved the final manuscript. All authors read and approved the final manuscript.

## Pre-publication history

The pre-publication history for this paper can be accessed here:

http://www.biomedcentral.com/1471-2377/14/99/prepub
